# Leisure-Time Physical Activity before and during Pregnancy Is Associated with Improved Insulin Resistance in Late Pregnancy

**DOI:** 10.3390/ijerph18094413

**Published:** 2021-04-21

**Authors:** Chenxi Cai, Zhengxiao Zhang, Samantha Mcdonald, Cody Strom, Rachel J. Skow, Linda E. May, Craig D. Steinback, Margie H. Davenport

**Affiliations:** 1 Program for Pregnancy and Postpartum Health, University of Alberta, Edmonton, AB T6G 2E1, Canada; ccai1@ualberta.ca (C.C.); rskow@ualberta.ca (R.J.S.); steinbac@ualberta.ca (C.D.S.); 2 Physical Activity and Diabetes Laboratory, University of Alberta, Edmonton, AB T6G 2E1, Canada; 3 Faculty of Kinesiology, Sport and Recreation, University of Alberta, Edmonton, AB T6G 2E1, Canada; 4 Women and Children’s Health Research Institute, Edmonton, AB T6G 2E1, Canada; 5 Alberta Diabetes Institute, Edmonton, AB T6G 2T9, Canada; 6 Department of Medicine, University of Alberta, Edmonton, AB T6G 2E1, Canada; zh16@ualberta.ca; 7 School of Kinesiology and Recreation, Illinois State University, Normal, IL 61790, USA; smmcdo4@ilstu.edu; 8 Department of Kinesiology and Sport, University of Southern Indiana, Evansville, IN 47712, USA; cstrom@usi.edu; 9 School of Dental Medicine, East Carolina University, Greenville, SC 27858, USA; MAYL@ecu.edu

**Keywords:** physical activity, pregnancy, insulin resistance, exercise

## Abstract

A total of 83 third trimester pregnant women were recruited to examine the role of pre-pregnancy versus late-pregnancy physical activity on maternal insulin resistance. Principal component analysis plots demonstrated a distinction between the high and low Homeostatic Model Assessment for Insulin Resistance (HOMA-IR) groups. The variation was driven primarily by exercise prior to and during pregnancy. Specifically, higher levels of physical activity prior to pregnancy was associated with a lower HOMA-IR and is not modified by other variables. Women who were active prior to pregnancy were more active during pregnancy. These results suggest that being active before pregnancy may be a good strategy for mitigating the risk of insulin resistance during late pregnancy.

## 1. Introduction

Recent meta-analyses have demonstrated that engaging in prenatal physical activity results in a 40% reduction in the odds of developing gestational diabetes mellitus (GDM) [1]. This reduction is believed to be related, at least in part, to exercise-induced improvements in insulin resistance. Even in the absence of overt GDM, subclinical insulin resistance is associated with adverse perinatal outcomes [2]. Given that maternal insulin resistance is a leading physiological candidate for gestational overgrowth [3], higher levels of maternal physical activity prior to or during pregnancy may help to improve maternal insulin sensitivity and β-cell function, resulting in a metabolically healthy pregnancy. A better understanding of the relationships between maternal physical activity prior to and during pregnancy, with a risk of insulin resistance, can inform the development of targeted behavioral interventions designed to promote optimal perinatal health outcomes. Hence, we evaluated the association between maternal physical activity prior to and during pregnancy and perinatal health outcomes.

## 2. Study Design

Healthy pregnant women in their third trimester were recruited at the University of Alberta. Written informed consent was obtained from all participants (Health Research Ethics Board, University of Alberta). Pre-pregnancy characteristics were collected through a Health History Questionnaire. Pre-pregnancy physical activity scores were self-reported using the Godin Leisure-Time Exercise Questionnaire (Godin Scale Score) [4]. Prenatal physical activity over the previous month was self-reported using the Pregnancy Physical Activity Questionnaire (PPAQ) [5]. A fasted blood sample was obtained to assess glucose (hexokinase, Seimens Advia 1800) and insulin (chemiluminescence microparticle immunoassay, Abbott Architect i2000) concentrations. Participants whose values exceeded the 75th percentile of the cohort were considered to have elevated but sub-clinical insulin resistance (HOMA-IR) [6]. Gestational weight gain was then classified as inadequate, adequate, or excessive based on the 2009 Institute of Medicine guidelines [7]. The participants were followed until delivery. Neonatal outcomes were categorized into preterm delivery, small-for-gestational age [8] or large-for-gestational age [9]. Residuals for all variants were plotted to check for normality. Unnormalized data were transformed before the statistical analysis. The multivariate data of health outcomes and contribution vectors were analyzed by principal component analysis (PCA) using the R package vegan [10]. Significant differences between groups were assessed via permutational multivariate analysis of variance. We determined associations between physical activity and health outcomes using logistic regression analysis. Differences of the means between the metabolically healthy vs. sub-clinical insulin resistance groups were compared using Student’s *t*-test. To assess the physical activity compositional interactions before and during pregnancy, Spearman’s correlation was performed. The *p*-values were adjusted using the Benjamini–Hochberg false discovery rate. All statistical analyses were performed using R, GraphPad Prism, and Stata.

## 3. Results

**Figure 1:**
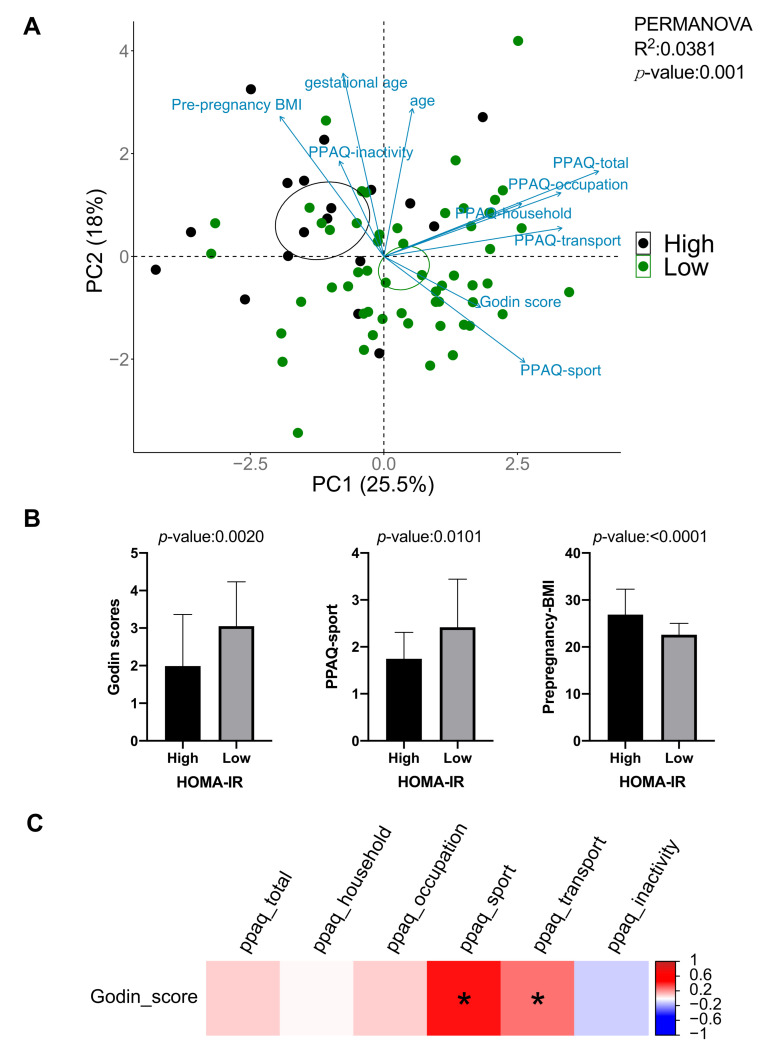
(**A**) Maternal physical activities and pre-pregnancy characteristics variables association with HOMR-IR in late pregnancy. A 2-dimensional scatter plot (PC1 vs. PC2) showing the variation of maternal physical activities and pre-pregnancy characteristics variables between high HOMA-IR group and low HOMA-IR group. The pre-pregnancy body mass index (BMI), exercise prior to pregnancy (Godin score), exercise during pregnancy (PPAQ-sport) variables are approaching 180° of separation, suggesting strong negative covariance between these variables contribute to the differentiation of high and low HOMA-IR scores. (**B**) Comparison of exercise prior to pregnancy (Godin score, cube root transformed), exercise during pregnancy (PPAQ-sport score, cube root transformed), and pre-pregnancy body mass index (BMI) between high HOMA-IR group and low HOMA-IR group. Statistical comparison between-groups were by unpaired *t*-test. (**C**) Associations between exercise prior to pregnancy and physical activities during pregnancy were assessed by Spearman rank correlation with FDR correction. * *p* < 0.05.

A total of 83 third trimester women with uncomplicated pregnancies were recruited. PCA plots based on the physical activities and pre-pregnancy characteristics variables (i.e., exercise prior to pregnancy, total physical activities during pregnancy, exercise during pregnancy, household-related physical activity during pregnancy, occupational activity during pregnancy, transportation activity during pregnancy, inactivity during pregnancy, age, pre-pregnancy BMI, race, gestational weight gain, parity, and gestational age), and demonstrated a distinction between the high HOMA-IR group (HOMA-IR > 2) and the low HOMA-IR group (*p* = 0.001, PERMANOVA; Figure 1A). The variation was driven primarily by exercise prior to pregnancy, exercise during pregnancy, and pre-pregnancy BMI. However, PCA ordination detected no significant differences in gestational weight gain (*p* = 0.226), large or small for gestational age (*p* = 0.737), birth mode (*p* = 0.835), or preterm delivery (*p* = 0.05). The *t*-test analysis was conducted to reaffirm the HOMA-IR results and showed that the low HOMA-IR group had a higher Godin score (*p* = 0.002) and PPAQ-sport score (*p* = 0.010), and a lower pre-pregnancy BMI (*p* < 0.001) compared to high HOMA-IR group (Figure 1B). Furthermore, the results of the univariate logistic regression showed that exercise prior to pregnancy (odds ratio [OR], 95% confidence interval [CI]:0.54, 0.36 to 0.83) and exercise during pregnancy (0.48, 0.25 to 0.91) resulted in a reduced odd of developing higher insulin resistance. To ascertain whether significant effects were independent of potentially confounding factors, multiple logistic regression models were performed using pre-pregnancy BMI, parity, and gestational age as covariates. Interestingly, the association between exercise prior to pregnancy and HOMA-IR was not influenced by any of these assessed covariates. However, physical activity during pregnancy did not modify insulin resistance after controlling for pre-pregnancy BMI. Our assessment of the association between exercise prior to pregnancy and physical activities during pregnancy showed that exercise prior to pregnancy was positively correlated with exercise during pregnancy (Spearman *r_s_* = 0.45, *q* = 0.0002; Figure 1C). This result suggests that having a physically active lifestyle prior to pregnancy is perceived to promote a woman’s physical activity during pregnancy.

## 4. Conclusions

To the best of our knowledge, this is the first cross-sectional study to show the independent effects of pre-pregnancy versus prenatal physical activity on insulin resistance and suggest more physical activity prior to pregnancy is associated with a lower HOMA-IR in late pregnancy. From a public health perspective, being active before pregnancy may be a good strategy for mitigating the risk of insulin resistance during late pregnancy. We acknowledge that sample size of current study was small, and a cross-sectional sampling has the impossibility of identifying causal relationships between the studied factors. Thus, well-designed interventions with a bigger sample size may be warranted.

## Data Availability

The study did not report any data.

## Abbreviations

GDM	gestational diabetes mellitus
PPAQ	Pregnancy Physical Activity Questionnaire
PCA	principal component analysis
